# Functional imaging of neuron–astrocyte interactions in a compartmentalized microfluidic device

**DOI:** 10.1038/micronano.2015.45

**Published:** 2016-02-29

**Authors:** Yandong Gao, Joey Broussard, Amranul Haque, Alexander Revzin, Tian Lin

**Affiliations:** 1 Department of Biomedical Engineering, University of California, Davis, CA 95616, USA; 2 Department of Biochemistry and Molecular Medicine, University of California, Davis, CA 95616, USA

**Keywords:** microfluidics, surface micropatterning, neurons, astrocytes, neuron-astrocyte interactions, biosensors

## Abstract

Traditional approaches in cultivating neural cells in a dish without orienting their interactions have had only limited success in revealing neural network properties. To enhance the experimental capabilities of studying neural circuitry *in vitro*, we designed an experimental system combining concepts of micropatterned surfaces, microfluidic devices and genetically encoded biosensors. Micropatterning was used to position neurons and astrocytes in defined locations and guide interactions between the two cell types. Microfluidic chambers were placed atop micropatterned surfaces to allow delivery of different pharmacological agents or viral vectors to the desired cell types. In this device, astrocytes and neurons communicated through grooves molded into the floor of the microfluidic device. By combining microfluidics with genetically encoded calcium indicators as functional readouts, we further demonstrated the utility of this device for analyzing neuron–neuron and neuron–astrocyte interactions *in vitro* under both healthy and pathophysiological conditions. We found that both spontaneous and evoked calcium dynamics in astrocytes can be modulated by interactions with neurons. In the future, we foresee employing the microdevices described here for studying mechanisms of neurological disorders.

## Introduction

Neurons communicate through highly orchestrated patterns of activity in both health and disease. These patterns include action potentials and inter- and intracellular signaling cascades. However, neurons are not the only important players in signal transmission. Astrocytes, a subtype of glial cells, extend thousands of fine processes that wrap around synapses and likely facilitate neurotransmission^1,2^. The notion of astrocytes as active players in communication within the nervous system is evidenced by recent reports of astrocytic calcium signaling leading to downstream regulation of interneuronal interactions in health and disease^3–6^. However, the role of astrocyte calcium signaling in the function of neural circuitry is poorly understood. Given the structural and functional complexity of neuron–astrocyte interaction *in vivo*, these phenomena are better studied *in vitro*, where the physiological configuration of neuron–astrocyte connectivity can be reassembled and their function studied individually in real time.

Genetically encoded calcium indicators (GECIs) with non-overlapping emission spectra are cutting-edge imaging tools for monitoring neuron–astrocyte interactions. The GECI toolkit includes the single-wavelength indicators GCaMP6^7^ and R-GECO^8^. Cells can be driven based on their specific pattern of gene expression to manufacture either of the proteins. Then, upon the influx of calcium (for example, following an action potential), GCaMP6 increases fluorescence emission with a green peak, whereas R-GECO increases fluorescence emission with a red peak. The spectral separation between the emission spectra of the two probes thus enables simultaneous imaging of calcium dynamics within distinct cell populations. In addition, because GECIs are generally viewed by means of microscopy, they allow the collection of detailed spatial information about recorded calcium dynamics.

Control over neuron and astrocyte interactions may be achieved using microfluidics. The field of microfluidics deals with manipulation of liquid in microscale channels and offers significant advantages over conventional cell culture methods in terms of reducing the use of costly reagents, media and cells^9–14^. In addition to these often-cited advantages, an exciting benefit of microfluidics is the ability to exercise spatiotemporal control over the cellular microenvironment^15^. The technology of microfluidics has seen extensive use in neurobiological research^16–18^. Inspired by the Campenot chamber^19^, various compartmentalized microfluidic devices have been fabricated for research in neuroscience and neurodegenerative diseases^5,20–33^. These devices employed the fluidic resistance of microgrooves^20–30^ or a valve-controlled soft wall^31–33^ to isolate neurons and astrocytes into individual chambers. Neural cells cultured in such compartmentalized microfluidic devices have been shown to remain viable and functional for prolonged periods of time^33,34^.

Surface patterning is another microfabrication-related technique applied in neurobiology for controlling the attachment of neural cells and for guiding extension of cellular processes^35–38^. Low-density neuronal cultures with single synaptic contacts have proven useful for unraveling synapse formation and neuronal signaling^39,40^. Patterning methods such as microcontact printing (μCP)^41–47^, micromolding in capillaries (MIMIC)^48–51^ and plasma-based dry etching^38^ have been utilized to pattern collagen I or poly-L-lysine (PLL) to form low-density neuronal networks.

Our study combined, for the first time, the following three technologies: (1) a compartmentalized microfluidic device to culture groups of neural cells in distinct and fluidically separated culture chambers, (2) surface patterning using polyethylene glycol (PEG)^52^, a nonfouling hydrogel, to define the cell depositing location and to guide neurite development, and (3) GECIs to provide a real-time readout of cellular activity. The experimental system integrating these three concepts was used for monitoring reciprocal interactions between astrocytes and neurons responding to stimulation with a pharmacological agent, glutamate. In the future, we envision utilizing this platform to unravel the contributions of specific cellular compartments to neurodegenerative and neurodevelopmental disorders such as Alzheimer’s disease, autism and Down syndrome.

## Materials and Methods

### Chemicals and materials

Phosphate-buffered saline (PBS; 1×) without calcium and magnesium, Dulbecco’s modified eagle’s medium (DMEM), 2-mercaptoethanol, laminin, poly-L-ornithine (PLO), L-glutamic acid, adenosine triphosphate (ATP), D-glucose, sodium bicarbonate (NaHCO_3_) and insulin were purchased from Sigma-Aldrich (St. Louis, MO, USA). Minimal essential medium (MEM), L-glutamine, penicillin–streptomycin, B-27 supplement, and Neurobasal-A (NBA) medium were purchased from Thermo-Fisher (Pleasanton, CA, USA). Transferrin was purchased from Millipore (Darmstasdt, Hesse, Germany). Negative photoresist (SU8-2050 and 2010) and developer solution (SU8-developer) were purchased from MicroChem (Newton, MA, USA). Polydimethylsiloxane (PDMS, Sylgard 184) was purchased from Dow Corning (Midland, MI, USA). Pyrex cloning cylinders (8 mm×8 mm) were purchased from Fisher Scientific (Pittsburgh, PA, USA). Glass coverslips (no.1, 24×60 mm) were obtained from VWR (West Chester, PA, USA). 3-Acryloxypropyl trichlorosilane was purchased from Gelest, Inc. (Morrisville, PA, USA). Polyethylene glycol diacrylate (PEG-DA, MW=6000) was purchased from SunBio Co. Ltd (Ansan, Gyeonggi, South Korea) and photoinitiator (Irgacure 2959) was purchased from Ciba (Basel, Basel-Stadt, Switzerland). The photomasks for electrodes and microfluidic channels were designed in AutoCAD and printed by CAD/Art Services (Bandon, OR, USA). Penicillin, streptomycin, trypsin-EDTA, Alexa Fluor 488, Alexa Fluor 546 and fluorescent ovalbumin were obtained from Life Technologies (Grand Island, NY, USA).

### PEG preparation and UV exposure

The PEG patterns were created by MIMIC using a PDMS mold 5 μm high^53^. A PEG hydrogel prepolymer solution was prepared by mixing 12 mg PEG-diacrylate (PEG-DA; MW=6000) with 98 μl 1× PBS and 10 μl 10% photoinitiator (Irgacure 2959). The glass coverslip (~150-μm thick) was first modified by acrylated silane, as previously described^53^. Silanization was necessary to ensure covalent anchoring of the hydrogel onto a glass substrate. The PDMS mold was brought into contact with a substrate to form a network of empty capillaries. A 2 μl PEG prepolymer solution was deposited on a punched port. A vacuum was applied on another port to aspirate the solution into the capillaries. Subsequently, the coverslip was flipped over and exposed under a ultraviolet (UV) source for 1 s using an OmniCure series 1000 light source inside an N_2_ bag. A PEG pattern remained on the coverslip after the PDMS mold was removed. The PEG pattern was avoided when, prior to bonding with the PDMS, the coverslip surface was carefully wiped with acetone.

### Device fabrication

The hydrogel micropatterns and PDMS microfluidic devices were fabricated using standard soft-lithography protocols^52,54^. Master patterns were created on four in wafers using negative-tone lithography. SU-8 2005 was used to create master patterns for hydrogel molding, and SU-8 2050 was utilized for making PDMS microfluidic devices. A prepolymer of PDMS was then mixed with a curing agent in a 10:1 ratio and poured over both molds. After degassing for 1 h, the PDMS layers were allowed to cure over their molds at 75 °C for 2 h. The solidified layers of PDMS were then peeled from their molds. A sharp, homemade needle puncher was used to make two small holes (~1 mm in diameter) at the diagonal corners of the PDMS mold for gel loading during MIMIC. In a similar manner, four holes (6 mm in diameter) were punched using a metal puncher to create media reservoirs in the microfluidic piece for inlets and outlets. After the PEG pattern was formed as described in the previous section, a piece of the PDMS with molded microfluidic channels and chambers was manually aligned onto the PEG pattern. Correct placement was ensured using a phase-contrast microscope. Subsequently, glass cloning cylinders (10 mm high, with an outer diameter of 10 mm) were placed into the inlet and the outlet holes were punched in the PDMS. The entire device was then placed into an oven and cured at 37 °C for 1 h. Pyrex cloning cylinders served as the liquid media reservoirs. The pressure difference between the reservoirs and the waste wells generated a continuous fluid flow through the microchannels and the cell chambers. During the curing process, approximately 200 μl of sterilized deionized water was loaded into each reservoir, maintaining a constant flow in the microfluidic channels to retain the hydrophilic nature of the PDMS surfaces.

### Surface coating

Neuronal compartments of the microfluidic co-culture devices were coated with 0.001 mg ml^−1^ PLO (diluted in DI water) for 12 h at room temperature and then washed in PBS for 10 min. This was followed by incubation for 12 h with 1 mg ml^−1^ PLL at room temperature. This was followed by another wash with PBS for 10 min. The astrocytic compartments were coated with 0.01 mg ml^−1^ collagen I solution for 12 h and washed in PBS for 10 min. Prior to seeding of the cells, the devices were sterilized by exposure to UV for 1 h.

### Neuron and astrocyte preparation

Neuronal^55^ and astrocytic^56^ cells were derived from hippocampi of P0 Sprague-Dawley rat pups as described previously. Purified astrocyte stocks were maintained in MEM supplemented with 28 mM D-glucose, 2.4 mM NaHCO_3_, 0.01% (w/v) transferrin, 2 mM L-glutamine, 0.0025% (w/v) insulin, and 50 μg ml^−1^ penicillin and 50 μg ml^−1^ streptomycin. All primary cell cultures were maintained in NBA supplemented with 2 mM L-glutamine, 50 μg ml^−1^ penicillin, 50 μg ml^−1^ streptomycin and 2% (v/v) B27 supplement inside microfluidic devices.

### Cell seeding and cultivation in microfluidic devices

After the liquid was removed from each reservoir, 10–30 μl of media with 3000 cells per μl was added to cloning cylinders serving as reservoirs^31,32^. The flow rate was adjusted by controlling the liquid head at the inlet and outlet media reservoirs^31^. After a desired flow rate (~50 μm s^−1^) was obtained, microfluidic devices were placed in a tissue culture incubator at 5% CO_2_ for 3 h at 37 °C to allow the cells to attach to the PLL-coated substrates. To wash away unattached cells, the inlet (loading) and outlet (waste) reservoirs were filled with 200 and 100 μl, respectively, and placed in an incubator for an additional 5 h. For routine culture, a continuous flow through the microchannels was maintained by adding 400 μl fresh media to the loading reservoirs and 100 μl to the waste reservoirs daily. The flow rate inside these devices was ~1 μl min^−1^ initially and slowed down to ~0.03 μl min^−1^ after ~5 h as the media levels in the reservoirs equilibrated. The rate of flow within the device was controlled between these values by varying the difference in volume applied to inlet and outlet wells. Given the microchamber volume of 0.6 μl, it took about 20 min to replace a whole chamber of media at this low flow rate.

### Integration of genetically encoded calcium indicators into neurons and astrocytes

GCaMP6 expression constructs were obtained from the Looger lab^7^, and those for R-GECO were obtained through Addgene (Plasmid #32444)^8^. Genes encoding these fluorescent reporters of neural and astrocytic activity were subcloned into a pAAV or pSIV backbone, with expression under the control of the pan-neuronal human *synapsin-1* promoter^57^ or the astrocyte-specific GfaABC_1_D promoter^58^. cDNA encoding reporters were packaged into live adenoassociated virus 2/1 (AAV2/1)^59^ or SIV-based lentivirus^60^. Recombinant AAV (rAAV) was purified via ultracentrifugation over a cesium chloride gradient, and recombinant lentivirus (rSIV) was prepared as a crude lysate.

Expression of the reporter proteins was then transduced in cells via viral introduction within 4–5 days after initial seeding. One microliter of virus-containing solution was added to 400 μl of appropriate culture medium. This mixture was pipetted into the loading reservoir, and 100 μl of fresh culture medium was introduced into the waste reservoir of a microfluidic device. Flow through the chambers was then allowed for 3–4 h, after which 200 μl of medium was transferred from the waste reservoir to the corresponding loading reservoir. This procedure was repeated three times to ensure sufficient viral incubation while maintaining a laminar flow rate that prevented the mixing of fluids between the chambers. The virus-containing culture medium was then discarded and replaced with fresh culture medium.

### Microscopy

Depending on which viral vector was utilized, the devices were prepared for imaging for 3 (for pSIV) or 5 (for pAAV) days after viral incubation. Culture medium was completely aspirated from the devices, and 100 μl Hank’s balanced salt solution (HBSS) was added to the loading reservoir. We applied the neurotransmitter glutamate to acutely stimulate cultures, as well as to model the longer term effects of excitotoxicity on neural cultures^5,61–63^. In these experiments, 100 μl of 100 μM glutamate in HBSS was infused into the inlet of the microfluidic chamber, thereby creating a 50 μM concentration of this stimulant.

The devices were imaged using a Zeiss 710 laser-scanning confocal microscope. For dual-color imaging (that is, both GCaMP6 and R-GECO), excitation was provided by the 488-nm light from an Argon laser and the 561-nm light from a diode-pumped solid-state laser. Excitation was directed to the stage by a 488/561 main dichroic beam splitter. The resultant emission produced by the sample was detected by two photomultiplier tubes with QUASAR detection units with bandpass set between 491 and 516 nm (for GCaMP6) and longpass set beyond 603 nm (for R-GECO).

### Image analysis

Cells were segmented for further analysis using manual thresholding of images in conjunction with the watershed segmentation algorithm in NIH ImageJ^64^. Then, regions of interest (ROIs) for further analysis were selected. In the case of neuronal data, the cell somata were manually encircled. For astrocytes, ROIs were automatically detected when the signal within a low-pass filtered image exceeded twice the baseline standard deviation (s.d.). For comparison of ROI size, any detected ROI connected in space and time was projected with an OR function onto a two-dimensional image. For all ROIs, Δ*F*/*F* was calculated as: $\left(F-F_{0}\right)/F_{0}$, where *F* is defined as the background subtracted, spatially averaged fluorescence intensity within the ROI and *F*_0_ is the average of the lower quartile of all *F* within a time series. Individual calcium events detected within each astrocyte’s ROI were fit with a Gaussian curve to allow calculation of the full-width at half maximum for each event.

## Results and Discussion

### Design and function of microsystems

**Figure 1:**
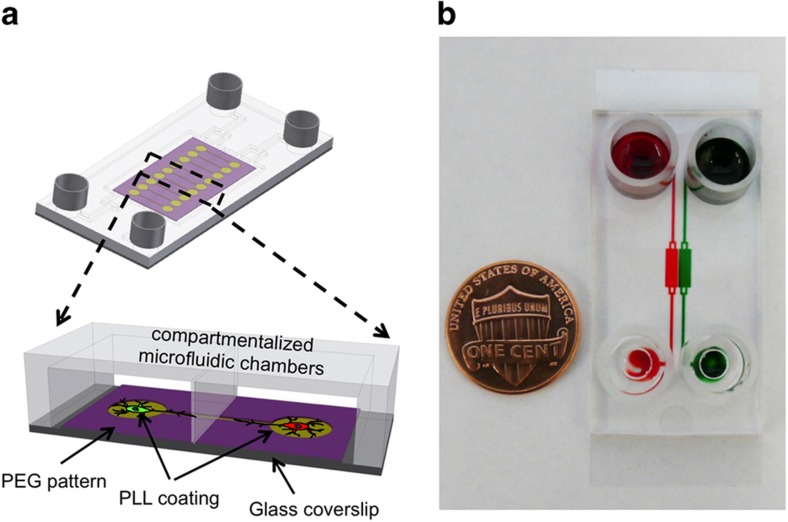
The design of compartmentalized microfluidic platform integrating surface patterning. (**a**) Schematics of the microfluidic device. (**b**) An assembled two-compartment platform. Food dyes were added to each compartment to show the channels and chambers. PEG, polyethylene glycol; PLL, poly-L-lysine.

The field of neuroscience has developed sophisticated optogenetics tools for triggering and monitoring the activity of specific neural cells or other cell populations^65,66^. However, standard neural cell culture approaches significantly limit the utility of optogenetics. The objective of this work was to develop a microfabricated platform for neural cell culture that will better leverage the full spectrum of capabilities offered by optogenetics. Specifically, our goal was to position neurons and astrocytes into distinct microfluidic compartments within a co-culture device. Glass substrates used for cell cultivation were micropatterned by a combination of PEG hydrogel micromolding and UV polymerization described by us previously^50–52,67^. Microfluidic channels were placed on top of micropatterned substrates, creating devices of the type shown in Figures 1a and b, in which neurons and astrocytes were separated by silicone rubber walls. Communication occurred via grooves molded in PEG hydrogel and running beneath the walls. Cell attachment sites and grooves for axonal guidance were coated with adhesion proteins such as collagen and PLL, and the regions of the glass substrate protected with PEG gel remained non-fouling and free of proteins.

This co-culture design was used to study the interactions between two populations of neural cells, such as neuron–neuron and neuron–astrocyte interactions (Figure 1b). As shown in Figure 1b, the microfluidic devices were small and portable. These devices were designed with biologists’ needs in mind: (1) the microfluidic channels sit atop thin coverslips suitable for live-cell imaging and high-resolution microscopy and (2) the reservoirs of this device hold enough culture media to maintain fluid flow over 24 h without pumps or tubing, making device operation simple and similar to multi-well culture plates.

### Limiting fluid flow and diffusion of molecules between adjacent compartments

One of the goals of this study was to selectively stimulate astrocytes vs. neurons co-cultured in a microfluidic device. To achieve this, our device needed to be designed so as to limit flow and diffusion between adjacent cell culture chambers while allowing neurons and astrocytes to contact each other. Controlling liquid exchange has previously been accomplished by means of a perfusion stream^28^ or valves situated between the adjacent compartments^31^. A simpler solution was recently described by Cohen *et al.* ^20^, who demonstrated that narrow and shallow grooves can be used to achieve near-complete fluidic isolation. In the present study, we implemented a similar design by employing 13 microgrooves, each 100-μm long, 10-μm wide and 5-μm high, to connect adjacent cell culture chambers of a microfluidic device.

**Figure 2:**
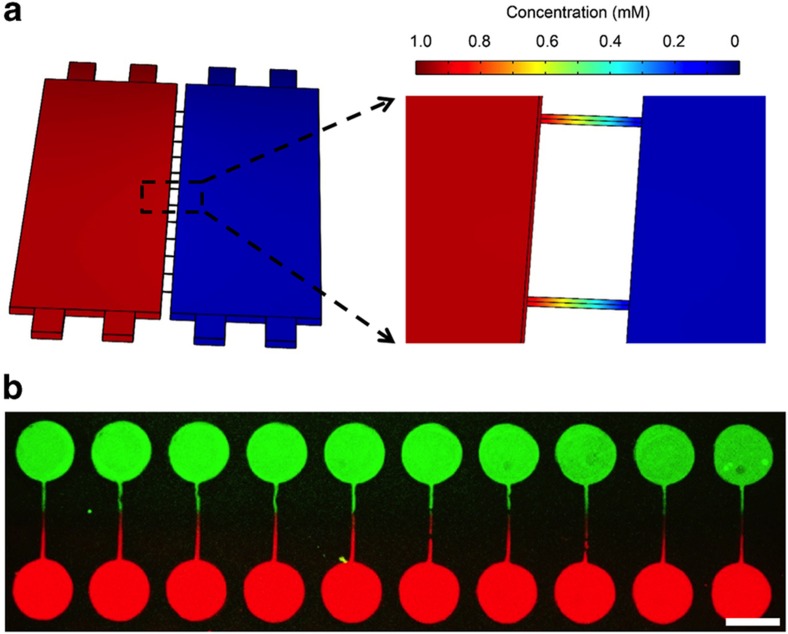
Fluidic isolation of the individual compartments in the co-culture device. (**a**) Stimulation result to show the effectiveness of fluidic isolation. The microgrooves defined two distinct microenvironments in each compartment. (**b**) Combined fluorescent images of a two-compartment device. The neuronal chambers were coated with two different fluorescent proteins. The scale bar represents 300 μm.

Numerical simulations were performed using COMSOL to validate that this design prevented convection and limited diffusion between adjacent cell culture chambers. Figure 2a shows the simulation results of concentration gradients in the two compartments of the microfluidic device. For this simulation, we set up a hypothetical scenario with the solute concentration being 1 and 0 mM in the left and right compartments, respectively. A diffusion coefficient of 5×10^–7^ cm^2^ s^−1^ was assumed—typical for proteins in an aqueous environment^68^. The flow rate chosen for modeling, 0.03 μl min^−1^, was the flow rate during most of the cell culture period. The simulation results in Figure 2a show that the concentration gradients within microfluidic compartments and grooves were quite steep. The concentration was ~0.5 mM at the entrance of the 100-μm-long groove and 0.07 mM at the end of the groove. Most importantly, the concentration of the model analyte at the sites of cell attachment, ~175 μm from the high-concentration compartment, approached zero. Therefore, modeling suggested that microgrooves can be used to effectively prevent diffusion of molecules between the compartments of a microfluidic device.

The experimental proof of limited diffusion between the compartments is shown in Figure 2b. In this experiment, inlet and outlet reservoirs were loaded with 400 and 100 μl, respectively, of media containing Alexa 488-labeled ovalbumin in the left channel and Alexa 546-ovalbumin in the right channel. As shown in Figure 2b, circular cell attachment sites and grooves in each compartment were stained with either red or green. No cross-compartment mixing of fluorescently labeled proteins was observed. This image also depicts the utility of the nonfouling hydrogel in confining protein deposition to the desired location on the culture surface. Thus far in this paper, we have described the fabrication of microfluidic devices and micropatterned surfaces. We have also demonstrated that the microfluidic devices were designed so as to limit cross-compartment convection and diffusion. In the next section, we demonstrate the utility of this microfluidic platform for investigating neuron–neuron and neuron–astrocyte interactions.

### Using microfluidic devices for cultivation and selective labeling of neurons with GECI

**Figure 3:**
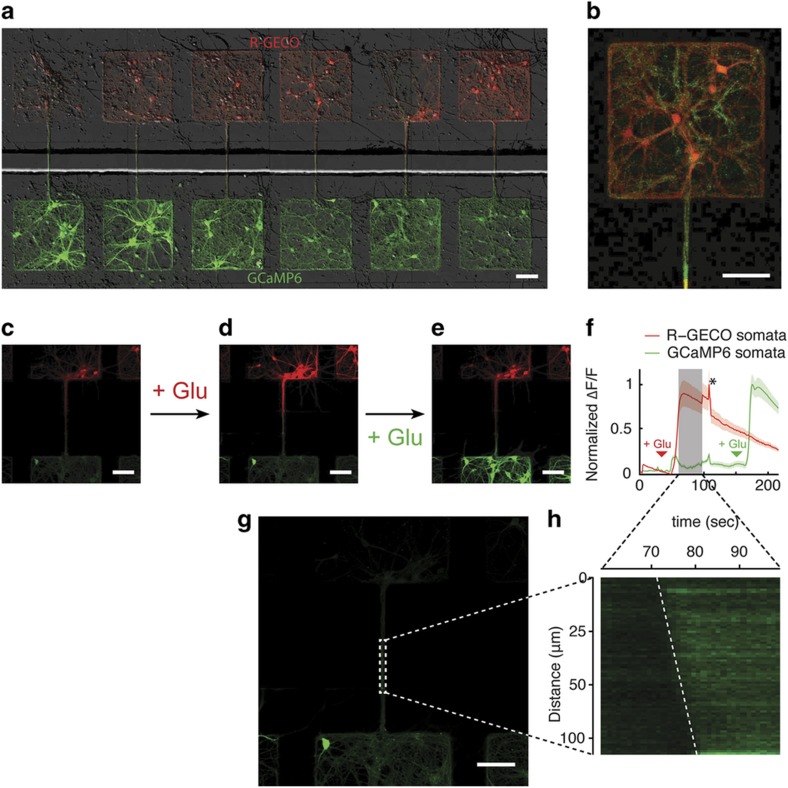
Virally infected two-color primary neuron response to glutamate. (**a**) A tiled image of neurons expressing R-GECO (top) and GCaMP6 (bottom). (**b**) An enlarged image from panel a demonstrating that axons from GCaMP6+ neurons enter the chamber containing R-GECO+ cells. (**c**–**e**) Sequential glutamate activation of R-GECO+ cells followed by activation of the GCaMP6+ cells. (**f**) Time course of cell responses shown in (**c**–**e**). Error bars show standard error of the mean at each time point. The asterisk indicates the point at which calcium influx showed a spontaneous increase on both sides of the device. The gray region of the graph indicates the portion of the time series, which is expanded in (**h**). (**g**) Spatial region of the GCaMP6+ axons expanded in (**h**). (**h**) A pseudolinescan demonstrating the calcium wave propagation speed and direction. All scale bars represent 100 μm.

Primary neurons were seeded into microfluidic compartments precoated with PLL. The cells became confined to the attachment sites defined within the PEG gel layer and began extending cellular processes. Prior to the time point at which the axons extended from one device compartment to the other (day *in vitro* (DIV) 4–5), high-titer viral samples with GCaMP6 or R-GECO coding under a synapsin promoter were introduced into each inlet reservoir. As shown in Figure 3a, neurons residing in separate compartments expressed either green (GCaMP6) or red (R-GECO) GECI. These results demonstrated that in a high-flow regime, small viral particles (~20 nm) did not travel down the grooves separating the two compartments. Figure 3a also indicates that both attachment of cells and extension of neurites could be effectively controlled by the micropatterned surfaces.

To further highlight the utility of this compartmentalized microfluidic platform, we delivered 50 μM glutamate first to R-GECO^+^ neurons and then to GCaMP6^+^ neurons. The glutamate evoked robust calcium transients as shown in the time-lapse images in Figures 3c–e. First, the fluorescence intensity became elevated in the R-GECO^+^ population as glutamate was introduced into the top chamber (Figure 3f). Due to the synaptic coupling between neurons in the two chambers, GCaMP6^+^ cells also exhibited increased fluorescence. The GCaMP6^+^ fluorescence increased further when glutamate was introduced to the chamber housing this cell population.

After application of glutamate to the R-GECO^+^ chamber, there was a rapid but short-lived rise in calcium that occurred on both sides of the device (the time point is indicated by an asterisk in Figure 3f). One possible explanation for this increase is a calcium wave that propagated along the GCaMP6^+^ axons (Figures 3g and h). In the case of the wave shown in Figure 3h, the speed of the wave propagation was 9.3 μm s^−1^.

PEG patterning of cell culture substrates allowed us to control the location of neuronal circuit formation. This enabled us to focus our microscopy studies on specific regions of the substrate where calcium signaling was expected to occur. Furthermore, these experiments demonstrated that our platform design provides fluidic separation between adjacent compartments and selective delivery of pharmacological compounds to the desired cellular compartment.

### Neuron–astrocyte interactions in microfluidic chambers

Next, we sought to establish that functional interactions between neurons and astrocytes could be achieved within our device. To do so, we imaged spontaneous calcium dynamics in the astrocytes co-cultured with neurons and the spatial distribution, amplitude and frequency of calcium signaling.

**Figure 4:**
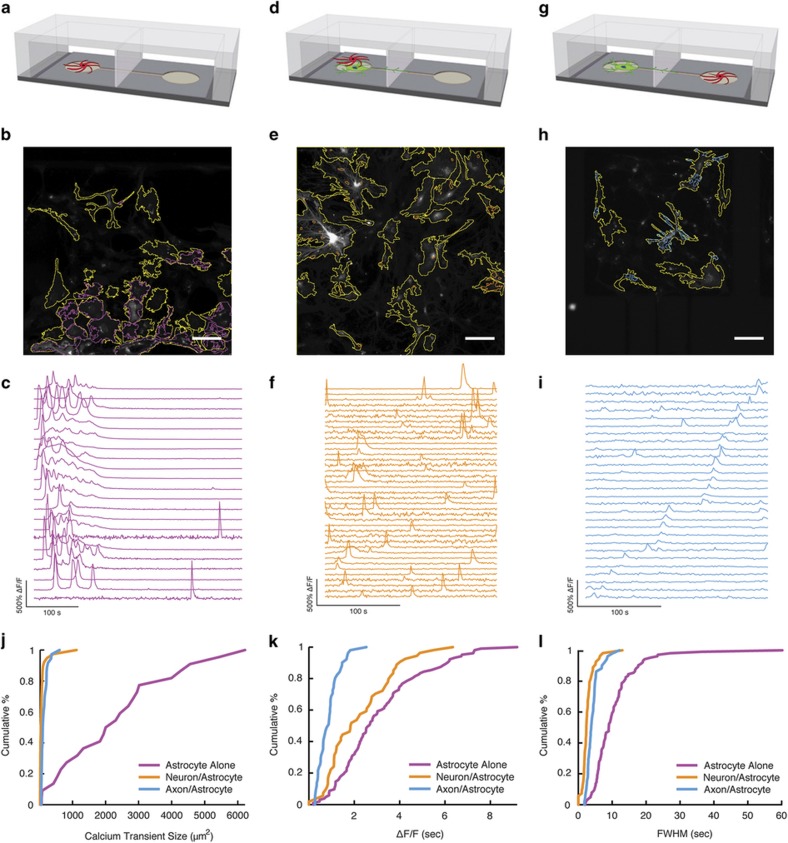
The presence of neurons or axons influences astrocyte calcium dynamics. Schematic demonstrating the three tested conditions: (**a**) astrocytes alone, (**d**) astrocytes seeded into the same chamber as neurons and (**g**) astrocytes seeded opposite neurons. Astrocytes in each condition (cell edges indicated by yellow ROIs) exhibit calcium transients of different size and frequency (magenta (**b**–**c**), cyan (**e**–**f**) and orange (**h**–**i**) ROIs) depending on the seeding condition. (**c**, **f**, **i**) Time course of calcium dynamics within ROIs identified as shown in panels **b**, **e**, and **h**. (**j**–**l**) Empirical cumulative distributions of (**j**) ROI size, (**k**) maximum Δ*F*/*F* per ROI and (**l**) dynamics of calcium events as measured by full-width at half maximum (FWHM) of ROIs shown in **b**, **e**, and **h**.

First, we established a baseline by seeding purified astrocytes into one chamber of a microfluidic device. Here these devices are referred to as A/- (Figure 4a). We found that spontaneous astrocyte calcium events were rare (two of eight imaging sessions in three devices) and propagated as waves across most of the cells within the field of view (Figures 4b and c).

Next, to assess the patterns of calcium signaling in the presence of both cell types, we co-cultured neurons and astocytes in microfluidic devices (Figures 4d–i). In these experiments, neurons and astrocytes were co-cultured either in the same microfluidic compartment (NA/-, Figure 4d) or in adjacent compartments (N/A, Figure 4g). To monitor the responses in each cell type, we labeled neurons with R-GECO driven by the neuron-specific promoter synapsin and astrocytes with GCaMP6 driven by the astrocyte-specific promoter GFAP. We found that neurons can modify the calcium dynamics in astrocytes (Figures 4d–i). For example, astrocytes co-cultured with neurons in the same chamber (NA/-) displayed spatially restricted microdomain calcium activity with higher frequency compared with astrocytes in monocultures (A/-; Figures 4f, j, and i). When neurons and astrocytes were co-cultured in adjacent compartments (N/A) with axons extending into the astrocyte chamber, the astrocytes displayed spatial and temporal calcium patterns similar to those observed in the NA/- condition (Figure 4i), although the amplitude of calcium was somewhat lower in the N/A devices (Figure 4k). Taken together, these experiments demonstrated that neurons can influence the spatial distribution and frequency of calcium signaling events in astrocytes.

**Figure 5:**
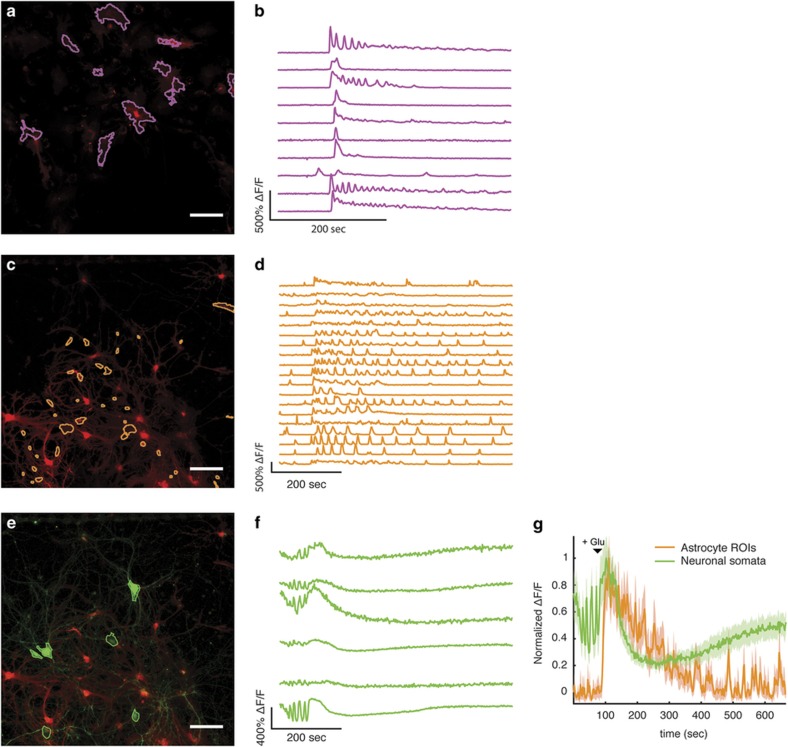
The presence of neurons influences astrocytic response to glutamate. (**a**) Astrocytes recorded in the A/- condition (**b**) along with time-course data from individual ROIs. (**c**) Astrocytes in the NA/- condition with an ROI color scheme as in Figure 4e–f. (**d**) Time-course data for individual ROIs is presented. (**e**) Composite image of neurons and astrocytes (from panel **c**) in the NA/- condition along with (**f**) time-course data from ROIs applied to neurons. (**g**) Averaged time courses of astrocyte and neuronal data as shown in panels **d** and **f** to allow comparison of the fluorescence response to glutamate. All scale bars represent 100 μm. Error bars are standard error of the mean calculated at each time point.

Given that astrocytes have been implicated in modulating the function of neural circuitry in neurological disorders^1^, we next sought to demonstrate the utility of our microfluidic device in studying astrocyte–neuron interactions under pathophysiological conditions. To model excitotoxic conditions, we continuously applied 50 μM glutamate to A- and NA/-cultures while imaging the calcium responses. In astrocyte monocultures (A-), this treatment triggered increased calcium transients and modulated their amplitude (Figures 5a and b). Astrocytes co-cultured with neurons (NA/-) displayed calcium transients with significantly increased frequency upon glutamate addition, followed by a slow decrease in frequency and amplitude (Figures 5c and d). Conversely, neurons displayed a sharp calcium flux in response to glutamate stimuli, followed by attenuation of calcium signals below the baseline level (Figures 5e and f). Interestingly, as the astrocytic calcium response began to taper, a slow rise in the calcium flux of neurons was observed (Figure 5g). Taken together, these results suggest that the presence of neurons or axons is sufficient to shape the manner in which astrocytes respond to their environment, under both normal and pathological conditions.

## Conclusion

Neural circuitry is shaped by complex interactions of pre- and post-synaptic neurons with astrocytes and other glial cells. There is increasing interest in understanding how interactions among these specific cell types shape neural circuitry in both the healthy and diseased nervous system.

The two-compartment microfluidic platform described here offers several advantages for the study of neuron–neuron or neuron–astrocyte interactions. This platform employs hydrogel microgrooves that serve the dual purpose of guiding axons and controlling cross-compartment diffusion of analytes. Diffusion of molecules between the adjacent compartments is limited, and selected groups of cells can be manipulated individually. In addition, the use of micropatterned surfaces allowed us to focus on selected areas of a cell culture surface. This is important for monitoring fast and complex processes, such as neuronal transmission, in which high-resolution microscopy has a limited field of view. In our system, it is possible to identify the areas where interesting events are most likely to occur and to prepare microscopy experiments accordingly. In addition, the micropatterning can further divide the neuronal network into smaller, isolated networks to enable parallel observations.

When cultured inside this microdevice, the neurons in one compartment extended neurites and formed functional interconnections with the neurons or astrocytes in the adjacent compartment. The neurons and astrocytes cultured in the microfluidic devices were healthy and could be stimulated or injured via selective delivery of molecules to the desired cellular compartment. In the future, this microfluidic device may be used to study the events in neurons that project axons into regions with pathophysiological extracellular states. These states include stroke and Alzheimer’s disease, in which excitotoxic levels of glutamate^69^ and beta amyloid peptide^70^, respectively, are released within the affected regions.

In addition, we are the first to report a calcium wave propagating in a retrograde direction along axons projecting into a high-glutamate environment. Such waves are known to cause a transient increase in the neural firing rate when propagated from the dendrites of neurons into the soma^71^. The speed of the waves observed in our culture system (~10 μm s^−1^) was much slower than calcium fluxes due to action potentials. On the basis of previous reports, it is likely that these slow-moving calcium waves are mediated by inositol triphosphate receptors (IP3R) in conjunction with calcium-induced calcium release^72,73^. Neural calcium waves are known to drive rapid responses such as axon withdrawal during early development^74^, as well as changes to neural excitability in adulthood^71^. However, the influence of retrograde calcium waves on neural computations has yet to be explored. Our approach thus serves to elucidate the functional consequences of this novel class of calcium waves.

This report of changes to astrocyte calcium signaling due to the presence of neuronal soma or axons is particularly timely. Recent work has uncovered a diversity of astrocytic calcium signaling occurring in situ and *in vivo* ^4,6,75,76^. Broadly, in intact and minimal preparations, it has been found that this diversity can be simplified by segregating calcium events by the volume of the astrocyte that they fill. More specifically, IP3R-dependent events have been shown to occupy a larger proportion of a given astrocyte volume than those that do not require access to subsurface calcium stores. These two classes of calcium events are in turn coupled to different downstream pathways^4,6^. Interestingly, we found that the presence of neurons or axons co-cultured with astrocytes shifted the distribution of calcium events in astrocytes in such a way that they occupied a smaller volume. Our system may thus allow further clarification of the contribution that calcium signals of different types make to astrocytic function.

Finally, we have shown with our approach that it is possible to simultaneously read out neural and astrocytic signaling in real time. Currently, it is known that astrocytes can reduce neural calcium flux through homeostatic control of the extracellular environment^77,78^, gliotransmitters^1,2^ and active secretion of neuroprotective factors^79,80^. However, it remains difficult to determine which mechanisms are paramount for neural protection in more intact preparations. In addition, very little is known about how neurons shape astrocytes’ response to excytotoxic conditions. The approach demonstrated here will be useful for addressing both questions.

The pharmacological manipulations that we employed to perturb cell function do not take full advantage of the temporal precision made possible by the use of GECIs as our readout. In particular, electrical stimulation would allow a higher degree of correlational fidelity between stimulus onset and resultant cellular calcium responses. Such stimulation techniques are compatible with the microfluidic devices employed in this study^81^. Incorporation of such technologies would further enhance the utility of our current design.

Overall, our approach allows direct, real-time observation of cellular function in isolated neuronal and astrocytic cultures. Although previous reports have modeled neural disease states using microfluidics, such studies relied on morphological changes observed at study end points^82^, potentially obscuring the time course within which the reported changes occurred. The combination of intracellular biosensors of calcium activity (GECIs) and microfluidics described in this study may help alleviate these issues.

## References

[bib1] Allen NJ , Barres BA . Neuroscience glia - more than just brain glue. Nature 2009; 457: 675–677. 19194443 10.1038/457675a

[bib2] Haydon PG . Glia: Listening and talking to the synapse. Nature Reviews Neuroscience 2001; 2: 185–193. 11256079 10.1038/35058528

[bib3] Sofroniew MV , Vinters HV . Astrocytes: biology and pathology. Acta Neuropathologica 2010; 119: 7–35. 20012068 10.1007/s00401-009-0619-8 PMC2799634

[bib4] Shigetomi E , Jackson-Weaver O , Huckstepp RT et al. TRPA1 channels are regulators of astrocyte basal calcium levels and long-term potentiation via constitutive D-serine release. The Journal of Neuroscience 2013; 33: 10143–10153. 23761909 10.1523/JNEUROSCI.5779-12.2013 PMC3682388

[bib5] Kunze A , Lengacher S , Dirren E et al. Astrocyte-neuron co-culture on microchips based on the model of SOD mutation to mimic ALS. Integrative Biology 2013; 5: 964–975. 23695230 10.1039/c3ib40022k

[bib6] Srinivasan R , Huang BS , Venugopal S et al. Ca(2+) signaling in astrocytes from Ip3r2(-/-) mice in brain slices and during startle responses *in vivo*. Nature Neuroscience 2015; 18: 708–717. 25894291 10.1038/nn.4001 PMC4429056

[bib7] Chen TW , Wardill TJ , Sun Y et al. Ultrasensitive fluorescent proteins for imaging neuronal activity. Nature 2013; 499: 295–300. 23868258 10.1038/nature12354 PMC3777791

[bib8] Zhao Y , Araki S , Wu J et al. An expanded palette of genetically encoded Ca(2)(+) indicators. Science 2011; 333: 1888–1891. 21903779 10.1126/science.1208592 PMC3560286

[bib9] Beebe DJ , Mensing GA , Walker GM . Physics and applications of microfluidics in biology. Annual Review of Biomedical Engineering 2002; 4: 261–286. 10.1146/annurev.bioeng.4.112601.125916 12117759

[bib10] El-Ali J , Sorger PK , Jensen KF . Cells on chips. Nature 2006; 442: 403–411. 16871208 10.1038/nature05063

[bib11] Meyvantsson I , Beebe DJ . Cell culture models in microfluidic systems. Annual Review of Analytical Chemistry 2008; 1: 423–449. 10.1146/annurev.anchem.1.031207.113042 20636085

[bib12] Mehling M , Tay S . Microfluidic cell culture. Current Opinion in Biotechnology 2014; 25: 95–102. 24484886 10.1016/j.copbio.2013.10.005

[bib13] Yeon JH , Park JK . Microfluidic cell culture systems for cellular analysis. Biochip Journal 2007; 1: 17–27.

[bib14] Dittrich PS , Manz A . Lab-on-a-chip: Microfluidics in drug discovery. Nature Reviews Drug Discovery 2006; 5: 210–218. 16518374 10.1038/nrd1985

[bib15] Young EW , Beebe DJ . Fundamentals of microfluidic cell culture in controlled microenvironments. Chemical Society Reviews 2010; 39: 1036–1048. 20179823 10.1039/b909900j PMC2967183

[bib16] Gross PG , Kartalov EP , Scherer A et al. Applications of microfluidics for neuronal studies. Journal of Neurological Science 2007; 252: 135–143. 10.1016/j.jns.2006.11.009 17207502

[bib17] Park JW , Kim HJ , Kang MW et al. Advances in microfluidics-based experimental methods for neuroscience research. Lab on a Chip 2013; 13: 509–521. 23306275 10.1039/c2lc41081h

[bib18] Millet LJ , Gillette MU . New perspectives on neuronal development via microfluidic environments. Trends in Neurosciences 2012; 35: 752–761. 23031246 10.1016/j.tins.2012.09.001 PMC3508261

[bib19] Campenot RB . Local control of neurite development by nerve growth factor. Proceedings of the National Academy of Sciences of the United States of America 1977; 74: 4516–4519. 270699 10.1073/pnas.74.10.4516 PMC431975

[bib20] Cohen MS , Bas Orth C , Kim HJ et al. Neurotrophin-mediated dendrite-to-nucleus signaling revealed by microfluidic compartmentalization of dendrites. Proceedings of the National Academy of Sciences of the United States of America 2011; 108: 11246–11251. 21690335 10.1073/pnas.1012401108 PMC3131323

[bib21] Ivins KJ , Bui ET , Cotman CW . Beta-amyloid induces local neurite degeneration in cultured hippocampal neurons: evidence for neuritic apoptosis. Neurobiology of Disease 1998; 5: 365–378. 10069579 10.1006/nbdi.1998.0228

[bib22] Park J , Koito H , Li J , Han A . Microfluidic compartmentalized co-culture platform for CNS axon myelination research. Biomed Microdevices 2009; 11: 1145–1153. 19554452 10.1007/s10544-009-9331-7 PMC2783938

[bib23] Park JW , Vahidi B , Taylor AM et al. Microfluidic culture platform for neuroscience research. Nature Protocols 2006; 1: 2128–2136. 17487204 10.1038/nprot.2006.316

[bib24] Peyrin JM , Deleglise B , Saias L et al. Axon diodes for the reconstruction of oriented neuronal networks in microfluidic chambers. Lab on a Chip 2011; 11: 3663–3673. 21922081 10.1039/c1lc20014c

[bib25] Ravula SK , Wang MS , Asress SA et al. A compartmented neuronal culture system in microdevice format. Journal of Neuroscience Methods 2007; 159: 78–85. 16876258 10.1016/j.jneumeth.2006.06.022

[bib26] Shi P , Nedelec S , Wichterle H et al. Combined microfluidics/protein patterning platform for pharmacological interrogation of axon pathfinding. Lab on a Chip 2010; 10: 1005–1010. 20358107 10.1039/b922143c PMC2867106

[bib27] Taylor AM , Blurton-Jones M , Rhee SW et al. A microfluidic culture platform for CNS axonal injury, regeneration and transport. Nature Methods 2005; 2: 599–605. 16094385 10.1038/nmeth777 PMC1558906

[bib28] Taylor AM , Dieterich DC , Ito HT et al. Microfluidic local perfusion chambers for the visualization and manipulation of synapses. Neuron 2010; 66: 57–68. 20399729 10.1016/j.neuron.2010.03.022 PMC2879052

[bib29] Yang IH , Gary D , Malone M et al. Axon myelination and electrical stimulation in a microfluidic, compartmentalized cell culture platform. NeuroMolecular Medicine 2012; 14: 112–118. 22527791 10.1007/s12017-012-8170-5

[bib30] Yang IH , Siddique R , Hosmane S et al. Compartmentalized microfluidic culture platform to study mechanism of paclitaxel-induced axonal degeneration. Experimental Neurology 2009; 218: 124–128. 19409381 10.1016/j.expneurol.2009.04.017 PMC4440669

[bib31] Gao Y , Majumdar D , Jovanovic B et al. A versatile valve-enabled microfluidic cell co-culture platform and demonstration of its applications to neurobiology and cancer biology. Biomedical Microdevices 2011; 13: 539–548. 21424383 10.1007/s10544-011-9523-9 PMC3085600

[bib32] Majumdar D , Gao Y , Li D et al. Co-culture of neurons and glia in a novel microfluidic platform. Journal of Neuroscience Methods 2011; 196: 38–44. 21185867 10.1016/j.jneumeth.2010.12.024 PMC3042731

[bib33] Shi M , Majumdar D , Gao Y et al. Glia co-culture with neurons in microfluidic platforms promotes the formation and stabilization of synaptic contacts. Lab on a Chip 2013; 13: 3008–3021. 23736663 10.1039/c3lc50249j PMC3712871

[bib34] Millet LJ , Stewart ME , Sweedler JV et al. Microfluidic devices for culturing primary mammalian neurons at low densities. Lab on a Chip 2007; 7: 987–994. 17653340 10.1039/b705266a

[bib35] Bernard A , Fitzli D , Sonderegger P et al. Affinity capture of proteins from solution and their dissociation by contact printing. Nature Biotechnology 2001; 19: 866–869. 10.1038/nbt0901-866 11533647

[bib36] Branch DW , Corey JM , Weyhenmeyer JA et al. Microstamp patterns of biomolecules for high-resolution neuronal networks. Medical & Biological Engineering & Computing 1998; 36: 135–141. 9614762 10.1007/BF02522871

[bib37] Offenhausser A , Bocker-Meffert S , Decker T et al. Microcontact printing of proteins for neuronal cell guidance. Soft Matter 2007; 3: 290–298. 10.1039/b607615g 32900145

[bib38] Rhee SW , Taylor AM , Tu CH et al. Patterned cell culture inside microfluidic devices. Lab on a Chip 2005; 5: 102–107. 15616747 10.1039/b403091e

[bib39] Kam L , Shain W , Turner JN et al. Axonal outgrowth of hippocampal neurons on micro-scale networks of polylysine-conjugated laminin. Biomaterials 2001; 22: 1049–1054. 11352086 10.1016/s0142-9612(00)00352-5

[bib40] Vogt AK , Wrobel G , Meyer W et al. Synaptic plasticity in micropatterned neuronal networks. Biomaterials 2005 May; 26: 2549–2557. 15585257 10.1016/j.biomaterials.2004.07.031

[bib41] Chang JC , Brewer GJ , Wheeler BC . A modified microstamping technique enhances polylysine transfer and neuronal cell patterning. Biomaterials 2003; 24: 2863–2870. 12742724 10.1016/s0142-9612(03)00116-9

[bib42] Fricke R , Zentis PD , Rajappa LT et al. Axon guidance of rat cortical neurons by microcontact printed gradients. Biomaterials 2011; 32: 2070–2076. 21167596 10.1016/j.biomaterials.2010.11.036

[bib43] Hart SR , Huang Y , Fothergill T . Adhesive micro-line periodicity determines guidance of axonal outgrowth. Lab on a Chip 2013; 13: 562–569. 23250489 10.1039/c2lc41166k PMC4123686

[bib44] James CD , Davis R , Meyer M et al. Aligned microcontact printing of micrometer-scale poly-L-lysine structures for controlled growth of cultured neurons on planar microelectrode arrays. IEEE Transactions on Biomedical Engineering 2000; 47: 17–21. 10646274 10.1109/10.817614

[bib45] Scholl M , Sprossler C , Denyer M et al. Ordered networks of rat hippocampal neurons attached to silicon oxide surfaces. Journal of Neuroscience Methods 2000; 104: 65–75. 11163412 10.1016/s0165-0270(00)00325-3

[bib46] Jun SB , Hynd MR , Dowell-Mesfin N et al. Low-density neuronal networks cultured using patterned poly-L-lysine on microelectrode arrays. Journal of Neuroscience Methods 2007; 160: 317–326. 17049614 10.1016/j.jneumeth.2006.09.009 PMC2767260

[bib47] Oliva AA , James CD , Kingman CE et al. Patterning axonal guidance molecules using a novel strategy for microcontact printing. Neurochemical Research 2003; 28: 1639–1648. 14584818 10.1023/a:1026052820129

[bib48] Vahidi B , Park JW , Kim HJ . Microfluidic-based strip assay for testing the effects of various surface-bound inhibitors in spinal cord injury. Journal of Neuroscience Methods 2008; 170: 188–196. 18314199 10.1016/j.jneumeth.2008.01.019

[bib49] Revzin A , Rajagopalan P , Tilles AW et al. Designing a hepatocellular microenvironment with protein microarraying and poly(ethylene glycol) photolithography. Langmuir 2004; 20: 2999–3005. 15875819 10.1021/la035827w

[bib50] Revzin A , Tompkins RG , Toner M . Surface engineering with poly(ethylene glycol) photolithography to create high-density cell arrays on glass. Langmuir 2003; 19: 9855–9862.

[bib51] Suh KY , Seong J , Khademhosseini A et al. A simple soft lithographic route to fabrication of poly(ethylene glycol) microstructures for protein and cell patterning. Biomaterials 2004; 25: 557–563. 14585705 10.1016/s0142-9612(03)00543-x

[bib52] You J , Shin DS , Revzin A . Development of micropatterned cell-sensing surfaces. Methods in Cell Biology 2014; 121: 75–90. 24560504 10.1016/B978-0-12-800281-0.00006-3

[bib53] Zhu H , Stybayeva G , Macal M et al. A microdevice for multiplexed detection of T-cell-secreted cytokines. Lab on a Chip 2008; 8: 2197–2205. 19023487 10.1039/b810244a

[bib54] Xia YN , Whitesides GM . Soft lithography. Annual Review of Materials Science 1998; 28: 153–184.

[bib55] Nunez J . Primary culture of hippocampal neurons from P0 newborn rats. Journal of Visualized Experiments 2008; 19: doi:10.3791/895. 10.3791/895 PMC2872976 19066540

[bib56] Mecha M , Iñigo PM , Mestre L. An easy and fast way to obtain a high number of glial cells from rat cerebral tissue: A beginners approach. Protocol Exchange 2011; 2011: doi:10.1038/protex.2011.218.

[bib57] Thiel G , Greengard P , Sudhof TC . Characterization of tissue-specific transcription by the human synapsin I gene promoter. Proceedings of the National Academy of Sciences of the United States of America 1991; 88: 3431–3435. 1849657 10.1073/pnas.88.8.3431 PMC51461

[bib58] Su M , Hu H , Lee Y . Expression specificity of GFAP transgenes. Neurochemical Research 2004; 29: 2075–2093. 15662842 10.1007/s11064-004-6881-1

[bib59] Grimm D , Kay MA . From virus evolution to vector revolution: use of naturally occurring serotypes of adeno-associated virus (AAV) as novel vectors for human gene therapy. Current Gene Therapy 2003; 3: 281–304. 12871018 10.2174/1566523034578285

[bib60] Hanawa H , Hematti P , Keyvanfar K et al. Efficient gene transfer into rhesus repopulating hematopoietic stem cells using a simian immunodeficiency virus-based lentiviral vector system. Blood 2004; 103: 4062–4069. 14976042 10.1182/blood-2004-01-0045

[bib61] Marks JD , Friedman JE , Haddad GG . Vulnerability of CA1 neurons to glutamate is developmentally regulated. Brain research Developmental Brain Research 1996; 97: 194–206. 8997504 10.1016/s0165-3806(96)00149-6

[bib62] Vergun O , Keelan J , Khodorov BI . Glutamate-induced mitochondrial depolarisation and perturbation of calcium homeostasis in cultured rat hippocampal neurones. The Journal of Physiology 1999; 519: 451–466. 10457062 10.1111/j.1469-7793.1999.0451m.x PMC2269520

[bib63] Gurkoff GG , Shahlaie K , Lyeth BG . *In vitro* mechanical strain trauma alters neuronal calcium responses: Implications for posttraumatic epilepsy. Epilepsia 2012; 53: 53–60. 10.1111/j.1528-1167.2012.03475.x 22612809

[bib64] Schindelin J , Arganda-Carreras I , Frise E et al. Fiji: An open-source platform for biological-image analysis. Nature Methods 2012; 9: 676–682. 22743772 10.1038/nmeth.2019 PMC3855844

[bib65] Palmer AE , Qin Y , Park JG . Design and application of genetically encoded biosensors. Trends in Biotechnology 2011; 29: 144–152. 21251723 10.1016/j.tibtech.2010.12.004 PMC3433949

[bib66] Broussard GJ , Liang R , Tian L . Monitoring activity in neural circuits with genetically encoded indicators. Frontiers in Molecular Neuroscience 2014; 7: 97. 25538558 10.3389/fnmol.2014.00097 PMC4256991

[bib67] Revzin A , Sekine K , Sin A . Development of a microfabricated cytometry platform for characterization and sorting of individual leukocytes. Lab on a Chip. 2005; 5: 30–37. 15616737 10.1039/b405557h

[bib68] Young ME , Carroad PA , Bell RL . Estimation of diffusion coefficients of proteins. Biotechnology and Bioengineering 1980; 22: 947–955.

[bib69] Xiong ZG , Zhu XM , Chu XP et al. Neuroprotection in ischemia: blocking calcium-permeable acid-sensing ion channels. Cell 2004; 118: 687–698. 15369669 10.1016/j.cell.2004.08.026

[bib70] Carter J , Lippa CF . Beta-amyloid, neuronal death and Alzheimer's disease. Current Molecular Medicine 2001; 1: 733–737. 11899259 10.2174/1566524013363177

[bib71] Hagenston AM , Fitzpatrick JS , Yeckel MF . MGluR-mediated calcium waves that invade the soma regulate firing in layer V medial prefrontal cortical pyramidal neurons. Cerebral Cortex 2008; 18: 407–423. 17573372 10.1093/cercor/bhm075 PMC3005283

[bib72] Ross WN . Understanding calcium waves and sparks in central neurons. Nature Reviews Neuroscience 2012; 13: 157–168. 22314443 10.1038/nrn3168 PMC4501263

[bib73] Jaffe LF , Creton R . On the conservation of calcium wave speeds. Cell Calcium 1998; 24: 1–8. 9793683 10.1016/s0143-4160(98)90083-5

[bib74] Yamada RX , Sasaki T , Ichikawa J . Long-range axonal calcium sweep induces axon retraction. The Journal of neuroscience : the Official Journal of the Society for Neuroscience 2008; 28: 4613–4618. 18448637 10.1523/JNEUROSCI.0019-08.2008 PMC6670433

[bib75] Shigetomi E , Kracun S , Sofroniew MV . A genetically targeted optical sensor to monitor calcium signals in astrocyte processes. Nature Neuroscience 2010; 13: 759–766. 20495558 10.1038/nn.2557 PMC2920135

[bib76] Agulhon C , Fiacco TA , McCarthy KD . Hippocampal short- and long-term plasticity are not modulated by astrocyte Ca2+ signaling. Science 2010; 327: 1250–1254. 20203048 10.1126/science.1184821

[bib77] Schousboe A , Waagepetersen H . Role of astrocytes in glutamate homeostasis: Implications for excitotoxicity. Neurotoxicity Research 2005; 8: 221–225. 16371316 10.1007/BF03033975

[bib78] Wang F , Smith NA , Xu Q et al. Astrocytes modulate neural network activity by Ca(2)+-dependent uptake of extracellular K+. Science Signaling 2012; 5: ra26. 22472648 10.1126/scisignal.2002334 PMC3515082

[bib79] Farahani R , Pina-Benabou MH , Kyrozis A et al. Alterations in metabolism and gap junction expression may determine the role of astrocytes as “good samaritans” or executioners. Glia 2005; 50: 351–361. 15846800 10.1002/glia.20213

[bib80] Cunha RA . Different cellular sources and different roles of adenosine: A1 receptor-mediated inhibition through astrocytic-driven volume transmission and synapse-restricted A2A receptor-mediated facilitation of plasticity. Neurochemistry International 2008; 52: 65–72. 17664029 10.1016/j.neuint.2007.06.026

[bib81] Morin FO , Takamura Y , Tamiya E . Investigating neuronal activity with planar microelectrode arrays: achievements and new perspectives. Journal of Bioscience and Bioengineering 2005; 100: 131–143. 16198254 10.1263/jbb.100.131

[bib82] Choi YJ , Chae S , Kim JH . Neurotoxic amyloid beta oligomeric assemblies recreated in microfluidic platform with interstitial level of slow flow. Scientific Reports 2013; 3: 1921. 23719665 10.1038/srep01921 PMC3667571

